# Hospital and Institutional News

**Published:** 1912-03-30

**Authors:** 


### HOSPITAL AND INSTITUTIONAL NEWS.

### THE MEDICAL PROFESSION'S UNITED FRONT.

At a fully attended meeting of representatives of the Medical Faculties of the English Universities, of the Royal College of Physicians of London, of the Royal College of Surgeons of England, and of the Society of Apothecaries of London on Thursday last week it was resolved unanimously: "That this Conference recognises that there is a remarkable unanimity of opinion within the medical profession as to the attitude which its members should adopt towards the working of the National Insurance Act of 1911. This Conference desires to place on record its general approval of the principles which inspire that attitude, and while conscious that there is some difference of opinion with regard to details, expresses its willingness to support the demand that these principles should be recognised by those who are responsible for the administration of the Act before medical practitioners consent to work under it." It is satisfactory to know from this resolution that the medical profession is still resolved upon a united front, and at this moment when public attention has been distracted from the profession's attitude on the Insurance Act such a reminder has added importance and value.

### HOSPITAL CLOSED THROUGH THE STRIKE.

Though the voluntary hospitals so far have been able to avoid the extreme measure of closing in consequence of the coal strike, one institution at least of the Metropolitan Asylums Board has been less fortunate. Owing to the urgent necessity for reducing the consumption of coal the Northern Hospital was recently closed and the patients were transferred to the Southern Hospital. The patients moreover were accompanied in this transfer by a large number of the Northern Hospital's staff. Some of them, however, are taking annual leave, and leave without pay, and many have been "lent" to other institutions of the Board. The hospital's committee of the board, of which Mr. J. Sprankling is chairman, has been compelled to adjust its arrangements through the strike in a novel and complicated manner.

### HAMPTON'S NEW COTTAGE HOSPITAL.

We are now in a position to supplement our recent announcement that a cottage hospital was to be given to Hampton, by disclosing the name of the generous donor, which has since been made public. At a meeting last week of the district council it was stated that Mr. T. Foster Knowles was responsible for the gift. Mr. Knowles has lived for many years in the parish, and desired to keep his anonymity at any rate till his return from abroad, but owing to negotiations for the purchase of the new hospital's site, his name has become public. The site which has been chosen by the three trustees, Mr. J. W. Restler, Mr. John Knowles (nephew of the donor), and Mr. Allbless, is on the west side of the Hampton Grammar School. The hospital will contain ten beds, and we understand that as soon as Mr. Knowles returns the trustees hope to begin work, in which case Mr. Allbless thinks that the hospital may be ready for use at the end of the year. It will be recalled that Mr. Knowles is to build, equip, and endow the new institution.

### THE LONDON MEMORIAL TO KING EDWARD.

The long controversy over the most appropriate form of memorial in London to the late King has a fresh interest to hospital workers from the fact that the statue on the Piccadilly site, which has only now to be accepted by the House of Commons, is to be embellished, according to Mr. MacKennal's description, by two supporting seated figures in bronze—"one representing Peace with emblems, and the other, symbolical of the hospitals, bearing the staff of Æsculapius." Whatever may be the criticisms of this site—and the likelihood of interfering with the present view of the Queen Victoria Memorial is a very obvious one—it is satisfactory to note the hospitals are to be associated with the memorial. By means of King Edward's Hospital Fund his name will be linked practically with our hospitals as long as the voluntary system endures. But it is a graceful complement to this to have the hospitals symbolically associated with London's memorial.

### THE KING EDWARD MEMORIAL AT LEEDS.

The Lord Mayor of Leeds, who is treasurer of the King Edward Memorial Fund, of which £40,000 out of the £150,000 aimed at has yet to be secured, has promised to help Mr. Middlebrook, the chairman, to make a great effort to obtain it. The need for this final effort was admirably summarised by Mr. Charles Lupton, honorary treasurer of the Leeds General Infirmary, who said: "There is now a disposition on the part of some people to think we have so much money that we cannot possibly want any more. As a matter of fact, an estimate of £150,000 was, if anything, too low for the work we want to do, and it is obvious that if we do not get the whole of that money we shall have to curtail the scheme. It would be a most lamentable thing if that were necessary. The scheme, as you will see when it comes out, is really a most excellent one, as it not only provides for the work we have to do to-day, but furnishes a method by which the infirmary can be extended as time goes on, and as our needs increase. We have had various schemes before us, and no other provides this advantage." This states the case very simply and correctly, and we hope that Leeds will realise the importance of raising the sum originally asked for.

### THE KING EDWARD MEMORIAL AT STOCKPORT.

A satisfactory achievement is announced at Stockport, in Lancashire. At the final meeting of the committee of the King Edward VII. Memorial Fund, the object of which was to raise the sum of £10,000 for the extension of the Stockport Infirmary, it was reported that the sum aimed at had been exceeded. The fund was opened last year, and the total amount raised was £10,909. This sum is to be handed over to the treasurer of the Infirmary for the extension alluded to, the understanding being that donors of £1,000 should be entitled to endow a full cot, donors of £500 a child's cot, and donors of £100 to become life vice-presidents of the Infirmary. Everyone interested in the raising of the standard of comfort and efficiency and accommodation, alike for patients and staff, will hear with much pleasure of the successful accomplishment of this object, in days when appeals rarely exceed the total aimed at.

### PENNY SUBSCRIBERS AT CHESTERFIELD.

Mr. E. C. Barnes, J.P., Chairman of the Board of Management of the Chesterfield and North Derbyshire Hospital, in moving the adoption of the report and statement of accounts last week, drew the attention of the meeting to the fact that the figures showing the number of occupied beds for 1910 and 1911 fully justified the additional accommodation. The determination of the Board to obtain the maintenance is most gratifying, the income almost meeting the expenditure. The bulk of the working-men now employed in the area are subscribing at the rate of 1d. per head per week. From this source alone £4,258 came last year, a rise of £2,346 since 1906. The support thus shown is highly praiseworthy to the locality and to the institution.

### NOTTINGHAM'S NEW EYE INFIRMARY.

At the opening of the new buildings of the Midland Eye Infirmary at Nottingham last week the Duchess of Portland performed the initial ceremony. The cost of the infirmary, of which Mr. A. Marshall is the architect, was given by the Secretary as £13,000. The Duchess appropriately recalled that Alderman J. P. Ford, who has presided over the committee for twenty-one years, fulfilled on that day the policy on which he and his committee had long set their hearts. And they certainly deserve congratulation for having opened the institution quite free from debt. It is interesting to add that one of the first patients received comes from Welbeck, and in honour of the opening it was decided to christen the ward in which the ceremony took place as the Portland Ward. There is no doubt that the occasion will be long remembered by Mr. Ford, for whom it was something more than a personal triumph, as all know who remember the pains which he and his committee have taken with the building scheme. The best reward that could be offered to them would be for the public to see that the heavy cost of maintenance is forthcoming.

### AN EARLY TEACHER OF WOMEN MEDICAL STUDENTS.

The retirement of Dr. Byron Bramwell, P.R.C.P.Ed., senior ordinary physician and lecturer on classical medicine at the Royal Infirmary, Edinburgh, apart from its intrinsic interest, is of importance, as he was the first physician who was placed in charge of the medical wards when these were opened to women medical students in 1892. His retirement is directly due to the operation of the rule whereby a physician's appointment is limited to fifteen years. Last year he added to his list of distinctions the LL.D. of St. Andrews University.

### THE ISLE OF MAN'S NEW HOSPITAL.

The last meeting in connection with the old Noble's Hospital in the Isle of Man has taken place, and very shortly, according to High-Bailiff J. S. Gell, the new hospital will be entered into. Noble's trustees are giving an endowment of £20,000 towards the new hospital, and, thanks to the special efforts of the enlarged committee which was appointed last year, and to the successful appeal in connection with the jubilee of the Isle of Man *Times*, an accumulated debt of £800 has been paid off, and a balance of £90 has been carried forward. In this connection it is instructive to add that for the first time the accounts have been presented on the uniform system. From the remarks of the speakers at the annual meeting it was evident that the new hospital, in the matter of building and equipment, has proved a bigger undertaking than was anticipated. The honorary secretary, Mr. R. D. Gelling, who is Secretary to the House of Keys, has been reappointed, and, as has been pointed out, he may look forward to an arduous year's work ahead. The committee is now considering how best to celebrate the opening.

### THE REBUILDING OF CHELSEA HOSPITAL FOR WOMEN.

The scheme for rebuilding the Chelsea Hospital for Women on the site of one and a quarter acres which, as we noted, the Earl of Cadogan gave some months ago, came up for discussion at the annual meeting on Tuesday. The site, which is between the Fulham Road and the King's Road, has been approved by the King's Fund, and the architect of the new buildings is Mr. Keith Young. The new hospital will contain seventy-six beds, in place of the fifty in the present building, and is expected to cost about £40,000. The present plan of small wards, which, though administratively expensive, is advantageous to the patients, will be maintained. Financially, much gratitude must be given to Mr. Dyer Edwardes, who, after having reduced the interest on the mortgage before, has now decided to forgo the mortgage altogether. The hospital will thus be able to dispose of its present site unencumbered. Moreover, £10,000 has been promised from the Zunz bequest. It is hoped that the new hospital will be finished in two years' time. Lord Castlereagh, who presided, in referring to the Insurance Act said that he hoped the King's Fund would consider it in the interval before its operation, so that a common policy might be followed. Mr. Henry E. Wright was re-elected honorary treasurer, but owing to his ill-health an acting treasurer has been appointed in the person of Mr. Sidney F. Hoffnung-Goldsmid.

### POOR LAW INFIRMARY EXTENSION AT PORTSMOUTH.

The new additions to the workhouse infirmary at Portsmouth, of which Lord Charles Beresford has now laid the foundation-stone, were begun last August. Two blocks are being built, one for men and one for women, and the accommodation will reach a total of 462 beds. An interesting addition is the phthisical wards, which will have 70 beds, and be placed not in a separate block but on a separate storey. If the buildings are finished in the early months of next year, as hoped, the completion of the 1898 scheme will have been achieved at a cost of £40,000. The medical superintendent of the workhouse infirmary is Mr. H. W. Morley, and the architect Mr. G. E. Smith, F.R.I.B.A. Every new infirmary of large size and every addition to existing buildings hasten the day when these Poor Law hospitals will be, as they ought to be, completely separated from the workhouses, and are therefore of great significance.

### AN AWKWARD SITUATION.

At the last meeting of the Bangor Guardians a curious mistake was reported. A tender for the erection of a new infirmary in connection with the workhouse, amounting to £15,500, had been formally accepted, and the contractor was asked for particulars to enable the Guardians to fill up a form required by the Local Government Board. In going through the papers an error involving a difference of £1,000 was discovered. The contractor presented the papers to the board for their consideration, and a long discussion followed. It was ultimately decided to invite new estimates. Undoubtedly this is the best way out of a difficult situation.

### NEW SECRETARY AT A LONDON HOSPITAL.

Mr. H. A. Dunn, the secretary of the Western Ophthalmic Hospital, has resigned after fourteen years' service. Mr. H. W. Burleigh has been appointed honorary secretary in his place. Mr. Burleigh is at present secretary of the Hospital for Epilepsy and Paralysis.

### THE LONDON MENTAL HOSPITALS COMMITTEE

The Mental Hospitals Committee of the London County Council has re-elected Mr. A. O. Goodrich to be its chairman during the ensuing year. Mr. Goodrich's reappointment is generally welcomed among all parties on the County Council. It is greatly to the credit of that body, however, that matters affecting the mental hospitals are never treated from any party standpoint. The Committee has also appointed its sub-committees for the various mental hospitals, and we note that Sir John McDougall, one of the few original members of the County Council who is still on that body, and who has throughout been associated with mental hospital work, is a member of every sub-committee.

### A MENTAL HOSPITAL APPOINTMENT.

Dr. Norcliffe Roberts, second assistant medical officer at Cane Hill Mental Hospital, has been appointed first assistant medical officer at Horton Mental Hospital, to fill the vacancy caused by Dr. Ogilvy's transfer to Long Grove Mental Hospital. Dr. Roberts, who is an M.D. of Durham, was formerly assistant medical officer at the West Riding Asylum, Wakefield.

### THE ASYLUMS OFFICERS BILL.

The Parliamentary Committee of the London County Council, reporting on Lord Wolmer's Asylums Officers (Employment, Pensions, and Superannuation) Bill, 1912, suggests that amendments be sought by the Council to provide that (*a*) not more than seven days out of the fourteen days of annual leave to be allowed to attendants and nurses after two years' service need be taken consecutively; (*b*) the liability to pay the contributions towards the pension of a superannuated officer who has formerly served in a Scottish parochial mental hospital will attach to the successors of the parish council in cases in which alterations of the constitution of the Council have taken place since the date of the former service of the superannuated officer; and (*c*) Clause 10 (1), dealing with the transfer to the Commissioners in Lunacy of the powers and duties of the Secretary of State under the Asylums Officers' Superannuation Act, 1909, shall be deleted, and the further powers of determining disputes conferred by Clause 10 (2) shall be vested in the Secretary of State instead of the Commissioners in Lunacy.

### LORD ROSEBERY AND THE KING'S FUND.

Presiding last week at the annual meeting of the Royal National Hospital for Consumption, Ventnor, Isle of Wight, Lord Rosebery took the opportunity to reiterate, in his own words, the complaint which annually fell from the chair at this meeting against King Edward's Hospital Fund. This complaint was that, while highly approving of the way in which their hospital was managed, the Trustees of the King Edward Fund did not see fit to give a grant to the Royal National Hospital. That complaint had in former years fallen on deaf ears, and he did not presume those ears would be any more receptive on this occasion than they had been before. Lord Rosebery was able to add that the annual subscribers showed no falling off, and that, though legacies had been scarcer than in the previous year, the total income for 1911 was £14,000. During the year two marble tablets have been put up to the memory of Queen Victoria and of King Edward VII.

### A HOSPITAL CHAIRMAN RESIGNS.

The first meeting of the newly elected board of management of the Royal Victoria and West Hants Hospital has been followed by the resignation of the chairman of the board, Dr. Roberts Thomson. Dr. Roberts Thomson has been connected with the hospital work for 40 years, and was, before the amalgamation, chairman of the board of the Bournemouth Royal Victoria Hospital, a post which he had held ever since its foundation in 1887. The new chairman of the joint board of the amalgamated hospital is Mr. D. H. W. Robson-Burrows. One of the vice-chairmen is Mr. E. H. Bellairs, who was previously chairman of the West Hants Hospital.

### "WORTH TWO REGIMENTS OF SOLDIERS."

Intelligence has been received by the Church Missionary Society of the death of two medical missionaries at Bannu, on the North-West frontier of India, from septicæmia. One of these is Dr. Pennell, a man whose name is known far and wide among those who take an interest in the turbulent tribes with whom our armies have had so often to contend. It is said that a commander of high rank in India once stated that Dr. Pennell was worth two regiments of soldiers on the frontier; and his name was a passport to a safe and hospitable reception by tribes whose first instinct is to waylay and murder any white man on sight. Dr. Pennell had a distinguished career at University College Hospital, whence he qualified in 1890; two years later he took up his life work on the North-West frontier. Here he soon established a reputation as a surgeon which spread far and wide among the tribesmen. This combined with a gift for languages and an enthusiasm for ethnology made him one of the foremost authorities on the wild and fierce peoples amongst whom he worked. His two books introduced him to a much wider circle at home, and the recognition of his work by the first class of the Kaisar-i-Hind medal was well merited. No information is yet to hand as to the nature of the septicæmia which has thus laid low both the medical missionaries at Bannu, and it is to be hoped that it may not turn out to be plague or any other form of epidemic specific septicæmia. At any rate Pennell and his colleague have died at their posts and have left behind them the imperishable monument of noble work.

### BOARDS OF GUARDIANS AND VOLUNTARY HOSPITALS.

A recent proposal to increase the subscription of the Clutton Guardians to the Paulton Cottage Hospital, near Bristol, was dropped in favour of an amendment put forward by Lord Strachie that a committee should be appointed to consider the question of subscriptions paid to hospitals by the Board, what advantage was derived from them, and whether it was advisable to increase or to decrease any of the subscriptions. The committee consists of Mr. Toke, chairman, Mr. W. W. Mattick, vice-chairman, Lord Strachie, Mr. W. Pooley, and Mr. G. Gould. It should be remembered that a subscription of five guineas to the hospital has been contributed annually by the Guardians for thirty years, and that at this moment there are in the hospital two patients who were in receipt of parish relief. One of these has been in the hospital since December 28, and the other entered on the 5th instant. The claim of the hospital on the Guardians is thus a prescriptive and very practical one, which does not need any research to establish.

### A RESIGNATION AT LEICESTER INFIRMARY.

Dr. F. M. Pope, F.R.C.P., the senior physician to the Leicester Infirmary, is to resign his position next month. By that date he will have completed twenty-six years of honorary service. Last week at the infirmary's annual meeting Dr. Pope was elected consulting physician, and the resolution to this effect was moved by Sir Edward Wood, Chairman of the board, and seconded by Sir Arthur Hazlerigg, Bart, Vice-Chairman. Every graceful emphasis, therefore, that tact could suggest was given to this resolution, the terms of which were thereby doubly enforced. Special attention, by the way, deserves to be given to the agenda paper, which is not only quite admirable in its type and appearance, but also is essentially an exact, if summarised, report of the proceedings of the meeting. This is only another instance of the thoroughness with which every detail of the work is carried out at Leicester, and the agenda paper of this annual meeting is a pattern which every hospital secretary in search of ideas for making such routine matters attractive may be recommended to consult.

### A REFORM LONG OVERDUE AT CHELTENHAM.

Speaking the other day at Cheltenham, Mr. Sydney Holland said with reference to the sleeping accommodation provided at the Cheltenham General Hospital, " I entirely agree with Sir Henry Burdett, who wrote in The Hospital, January 15, 1910, ' the sleeping accommodation at this hospital for its nursing staff is a discredit to the Board of Management, for it is undoubtedly the very worst to be found anywhere in the South and West of England.' " This Report, like others of the series, is thus shown to have borne the test of time by being corroborated two years later by independent testimony. But this is not the point which we wish to emphasise. The important fact is that two years have been allowed to pass by those responsible for the well-being of the Cheltenham General Hospital without any effort being made to improve the glaringly inadequate accommodation for the nurses, which was exposed in January 1910. Want of funds will be, no doubt, the excuse put forward, but this cannot be accepted, since no appeal has been made to the public to provide money for this purpose. This, however, is said to be forthcoming, and though most discreditably late in the day, we hope it will be presented as forcibly as the need for it demands, so that the public of Cheltenham may be convinced of the necessity for removing the stigma which has been allowed to attach to one side of the hospital's work, and to impair its efficiency.

### THE CHARITY COMMISSIONER'S INQUIRY AT SPALDING.

The proceedings before the Charity Commissioner, Mr. G. W. Wallace, in the inquiry he held at Spalding at the beginning of the month, as we intimated last week, form interesting reading for hospital managers. The Johnson Hospital, which is the most recently endowed hospital in the country, is managed by a number of trustees, many of whom are clergymen of the Church of England, of whom the present chairman is one. The inquiry was rather thinly attended; several of the trustees were present, some of whom interpolated remarks as the inquiry proceeded. Thus the Chairman commenced with a statement that the original report by our Commissioner on the Johnson Hospital (see The Hospital for November 18, 1911, p. 183) was one which the trustees had treated with silent contempt. Then the Charity Commissioner elicited the fact that changes had been initiated since the first report appeared in regard to the internal, hygienic, and decorative condition of the hospital. The chairman then elaborated with some of his fellow trustees the amazing doctrine that these trustees held a sacred trust, quite oblivious of the fact, as the report and evidence showed, that, whatever they might call it, their ideas of fulfilling so sacred a duty have, to say the least, left something to desire for some years past.

### A CLOSED DOOR.

The board of trustees at the present time is a close body, and the enlightened opinion of Spalding has previously, and did in the course of the inquiry, demand, that public bodies and all shades of religious and public opinion should be represented on the board of trustees. The answer given to this reasonable contention was to utter firmly the words "sacred trust," to express sympathy with the unrepresented interests, and regret that the sacred trust blocked the way to their representation. Further, and still more wonderful, the chairman maintained that as the monies left in trust had come from two residents in Spalding, although the hospital is in need of an increased income, the people resident in the parishes which have specially benefited by the money left to maintain the hospital should not, for this very reason, be pressed to give subscriptions and donations to the hospital's funds.

### TALKING TRUSTEES.

This extraordinary suggestion was enforced by the claim that the trustees had talked over matters for a couple of years without doing anything. This contention will convey to every manager of a modern hospital the real causes to which the present condition of the Johnson Hospital may greatly be attributed. If the people of Spalding want to have a hospital worthy of the name, and to make it available for all classes of the community, from those who can pay a remunerative rate to the humblest citizens, and to ensure its economical and efficient administration for the future, they will support the findings of the Charity Commissioners. Thus they may secure the introduction of sufficient independent representatives of all interests upon the board of trustees to ensure that the management in future shall not longer remain somnolent and ineffective.

### MR. C. B. LOCKWOOD'S RETIREMENT.

A vacancy has been declared on the senior surgical staff at St. Bartholomew's Hospital, due to the retirement of Mr. C. B. Lockwood. The senior assistant surgeon is Mr. McAdam Eccles, and if he receives his promotion a vacancy will thus be created for an assistant surgeon. Mr. Lockwood has not, of course, completed the full term of office to which he is entitled; nevertheless he has rendered yeoman service to St. Bartholomew's, to the school there, and to surgery as a science. His first qualification dates back to 1878, and his connection with St. Bartholomew's has been practically continuous ever since. He has been Hunterian Professor three separate times, President of the Medical Society of London, and a member of the Council of the Royal College of Surgeons. Mr. Lockwood's contributions to medical literature have been very numerous, and on widely different topics; yet it is mainly in connection with the development of Listerian principles in operative surgery and with the treatment of traumatic infections that his work will be best remembered.

### SUDBURY'S NEW ISOLATION HOSPITAL.

On the Clay Pits, Waldingfield Road, Sudbury, a new isolation hospital has been opened by Mr. T. Miles Braithwaite, Mayor, who recalled the fact that in these same clay pits the borough council was ordered to build "pest houses" at the time of the Great Plague two hundred and fifty years ago. Mr. W. L. Tait, the Borough Surveyor, was congratulated by the Mayor on his share of the work. There is accommodation for twelve beds, and the total cost is £600. The hospital contains two wards, kitchen, nurses' room, bathroom with geyser, and discharging room, and is situated on the outskirts of the town, in grounds which have been laid out for future attractiveness.

### A GENEROUS COTTAGE HOSPITAL CHAIRMAN.

Mr. A. Grant-Meek, Chairman of the Devizes Cottage Hospital, has been receiving local thanks for the important part he has played in the enlargement scheme. The accommodation is being increased from seventeen to twenty-one beds, and a children's ward is being added, while the matron's and her staff's quarters are being improved. Canon Gardiner affirms that the scheme could not have been carried through if it had not been for Mr. Grant-Meek, who "had, as a matter of fact, given just about one-half of the total amount contributed." This is a good instance of the keenness with which the best cottage hospital chairmen devote themselves to their institutions.

### THE COST OF LONDON'S SMALLPOX HOSPITALS.

A hot debate on the cost of London's smallpox hospitals took place at a meeting of the managers of the Metropolitan Asylums Board last Saturday. Canon Sprankling presented a report from the Hospitals Committee which recommended that Long Reach Hospital, which was built of wood ten years ago, should be gradually rebuilt in permanent material. Mr. Helby remarked that the time had arrived to stop the enormous expenditure upon the Long Reach Hospital where the heating and lighting alone last year cost £4,000. They also had the Orchard Hospital which had never been used and on which the capital charges amounted to £115,000. During the last eight years the average number of patients accommodated in the smallpox hospitals was 10.3 per year while the cost had been £183,000. Mr. Helby said that the expenditure on the hospitals had become a scandal; but another member, Mr. Ecroyd, urged that the expenditure on maintenance was a form of insurance and the fact that the small-pox hospitals were empty was a matter for congratulation. It was eventually decided to rebuild two of the wards at Long Reach Hospital in permanent material. There is a good deal to be said for Mr. Ecroyd's view, but the question still remains how much it is statesmanlike to spend on this insurance.

### GIFT OF A CONVALESCENT HOME TO ST. GEORGE'S HOSPITAL.

By the will of Mrs. Margaret Beet Swaine, who died early this year, her residence of The Highlands, Bexhill-on-Sea, is left to St. George's Hospital to be used as a convalescent home. The residue of her estate (which is understood to be in the neighbourhood of £10,000) is also left to the hospital for the endowment of this home. Failing the acceptance of the offer of the convalescent home, the trustees have power to distribute the whole legacy among such hospitals (including St. George's) as they may think fit. It is, we may suppose, unlikely that the authorities of St. George's will turn up their noses at this handsome bequest, though convalescent homes at a considerable distance from the parent institution are often difficult and troublesome to manage. Further, St. George's already possesses at Wimbledon a very large and well-endowed home, called the Atkinson-Morley in commemoration of its founder. This home is used chiefly for relieving the pressure on the wards of the hospital by transferring for the later portion of their recovery period those patients who would otherwise stay longer at Hyde Park Corner. That is to say, it is not used as a sanatorium for infective (*e.g.*, tuberculosis) patients. This is, of course, the proper function of such a home. In the case of Bexhill, however, the expense of sending patients there will probably prohibit its use for those who merely require a week or a fortnight of rest before returning to work; and it may seem advisable to utilise it instead for cases of a kind which are quite properly refused at the Atkinson-Morley, such as surgical tuberculosis, convalescence from serious infective diseases such as typhoid fever, cases of recovering endocarditis from rheumatism, and so on. The endowment provided is not any too large for its purpose, and additions to it would be a worthy object for any testators who are thinking of making charitable dispositions of their estates.

### NEW COTTAGE HOSPITAL SECRETARY.

Mr. C. R. Scrase Dickins, J.P., who has accepted the Horsham Cottage Hospital's presidency for another year, has drawn attention to the fact that the institution's financial prosperity was suffering from a belief that it did not need increasing support. He pointed out that since the number of beds has been increased the necessity for further subscriptions was urgent. Mr. A. O. Streatfield is the new honorary secretary, and it is to be hoped that his coming year of office may be marked by public recognition of the fact that the cost of maintenance is seriously increased by the additional beds which have been added.

### SOME NEW HOSPITALS IN GERMANY.

Among the newly designed hospitals which have recently been opened abroad, and are of special interest to the architect or hospital administrator, by reason of their presenting special and noteworthy features in construction, several stand out prominently. We have already referred to the designs for the new Jewish hospital to be built at Berlin, which promises to be one of the most instructive German hospitals. The newly constructed municipal hospital at Fisheln is interesting because of the large garden which has been made a feature, and also because of the fact that the regulations allow patients to choose their own doctor—a striking innovation in municipal hospitals, the results of which will be followed with some anxiety by its private competitors. The operation rooms at this institution are remarkably well arranged, and exhibit in a fine manner the commonsense principles of simplicity in construction which are so marked a feature in modern German hospitals. At Menden, in Westphalia, a small hospital, the plans of which show several novel points in regard to the arrangement of the ward annexes, has been opened. At Berlin the new Charité buildings are nearing completion, and present several equally interesting features, chiefly, however, from an architectonic point of view. Other new plans that may be mentioned are those of the Denklingen Sanatorium near Cologne, the new Gynæcological Clinic at Dantzig, and the extension of the Brandenburg municipal hospital. All these show very clearly the desire of modern hospital constructors to economise construction outlay by writing down expenses on unnecessary addenda. It is to be noticed, for instance, that in nearly all considerable attention has been devoted to the arrangement of the annexes and of the special departments.

### THIS WEEK'S DRUG MARKET.

Business in the drug market has been without improvement; in fact, it has been duller than last week. The most important event is the withdrawal, as from April 6, of two important firms from the Glycerin Convention. Those two firms gave notice to withdraw some time ago but changed their intentions. Now, however, they have changed again, and the result will probably be competition in the glycerin market, and therefore lower prices. The price of cocaine has been reduced. Oil of cloves is rather dearer. Chamomiles and senega are tending upwards in price, while serpentaria and damiana are tending lower. There is little change in the position of cod-liver oil, but there is still a tendency towards lower prices. The prices of opium and morphine are still tending slightly downwards, but there is no alteration in the quotations for codeine.

### TO OUR READERS.

Contributions are specially invited from any of our readers to these columns. They should deal with topical subjects and news. They must be authenticated for the information of the Editor only. The minimum payment if published is 5s. There is no hard-and-fast rule as to space, but notes of about twenty lines in length are preferred.

